# Regulatory Lag, Regulatory Friction and Regulatory Transition as FinTech Disenablers: Calibrating an EU Response to the Regulatory Sandbox Phenomenon

**DOI:** 10.1007/s40804-021-00217-z

**Published:** 2021-07-21

**Authors:** Deirdre Ahern

**Affiliations:** Professor in Law, Director of Technologies, Law and Society Research Group, School of Law, Trinity College Dublin, Dublin, Ireland

**Keywords:** FinTech, Regulatory sandbox, EU financial services law, Digital finance, Initial coin offerings, Crypto-assets, Crowdfunding

## Abstract

With transformative evolution involving crypto-assets, machine learning applications and data-driven finance models, complex regulatory and policy issues are emerging. Inadequate frameworks in FinTech markets create regulatory friction and regulatory fragmentation. These limitations continue to feature when piecemeal regulatory transition occurs. The danger of EU Member States being left behind in the FinTech innovation race if the regulatory landscape is cumbersome or incomplete for new business models is real. Regulatory lag and regulatory friction also act as a ‘disenabler’ for ease of cross-border FinTech trade in the EU. This article critically engages with the manner in which the regulatory sandbox has rapidly gained critical mass in Member States as a valuable adaptive measure supporting a route to market for FinTech entrepreneurs. Against the backdrop of the European Commission’s Digital Finance Strategy, the article further advances scholarship on FinTech in the EU by probing the EU’s resulting regulatory dilemma, undertaking a systematic evaluation of the continuum of complex policy options available to the European Union in response to the spreading regulatory sandbox phenomenon.

## Introduction

Finance and technology have proved to be partners made in heaven as the global FinTech landscape evolves at an exponential pace.^1^ Digitalisation is rapidly changing how business is done, transforming capital markets and making financial services more easily accessible and more easily executed. New business models and delivery methods have huge potential for revenue growth and are disrupting and superseding established ways of doing business. New methods of payments and finance have emerged. Big data analytics, cloud computing and artificial intelligence (‘AI’) are being harnessed. Moreover, the COVID-19 pandemic has boosted demand for access to digital financial services and the power of challenger banks to increase market penetration. There is a manifest need for the EU institutions to concretely respond to the opportunities and challenges that the technological advances of the fourth industrial revolution brings and to calibrate regulation and authorisation regimes so as facilitate appropriate market entry while guarding against risk.

There is a risk of EU Member States losing out on FinTech market share if the regulatory landscape is too difficult to manoeuvre or imposes a disproportionate regulatory burden on FinTech market actors, disenabling rather than facilitating FinTech. Regulatory and supervisory responses to financial applications of emerging technologies are far from crystallised given that their very novelty means that the risks are not fully captured. Consequently a comprehensive credible regulatory single market strategy for FinTech has not yet emerged. Nonetheless, some Member States have already individually carved out a FinTech niche and positioned themselves ahead of the curve as new business models fuelled by complex technological innovation are constantly pushing boundaries.

The regulatory sandbox forms one piece of a hugely challenging regulatory jigsaw puzzle whose enigmatic parts are not static, but evolving. Its invention responds to the need for regulators to gain first hand understanding of new technological developments rather than risking an inept approach to regulating emerging technologies when business models and risks are not yet fully bedded down and understood. A type of experimental governance first seen in 2016 as a mechanism for small-scale supervised testing of new FinTech services under the eye of a regulator, the regulatory sandbox is now an accepted tool in the regulatory toolbox around the globe.^2^ Adoption of the regulatory sandbox by regulators can be characterised as a stopgap measure in the absence of a *sui generis* regulatory regime tailor-made for FinTech. Given that it functions as a work-around to facilitate market entry by FinTech operators, understandably a growing number of EU Member States are falling under the thrall of the regulatory sandbox. The impact of this regulatory phenomenon gaining critical mass within the European Union should rightly command both regulatory and scholarly attention. Furthermore, an EU-centred view of the place and operation of regulatory sandboxes within the context of optimal regulatory approaches to FinTech is needed. As the EU begins to address questions of FinTech regulation in earnest, an evaluation of sandbox design matters is critical. A dilemma arises for the EU as to what extent the EU should immediately engage with the regulatory sandbox in the short-term versus concentrating its resources on the painstaking and drawn-out work of devising hard law regulation for FinTech for the medium haul. However, the choice does not have be a binary one. Pursuing both options simultaneously would deliver an agile twin track strategy to address regulatory unwieldiness and regulatory lag. The analysis undertaken here takes place in a context where the EU institutions are running their rule over the regulatory sandbox phenomenon following extensive stakeholder engagement and the adoption in late 2020 by the Commission of its Digital Finance Package. This includes the Digital Finance Strategy^3^ which demonstrates a potentially pivotal commitment to the development of a framework for cross-border testing in the EU.

Inadequate and embryonic EU financial services frameworks create both regulatory friction and regulatory fragmentation in FinTech markets that hinder cross-border scaling. This limitation continues to apply as regulatory transition occurs where emerging elements of bespoke FinTech regulation added to the regulatory mix are piecemeal and disjointed rather than comprehensive. Accordingly, Sect. 2 begins by setting the commercial and regulatory background through outlining FinTech models and mapping the regulatory and policy landscape within which they currently interface in the European Union. Flowing from this discussion, Sect. 3 contextualises the origination and nature of the regulatory sandbox phenomenon in regulatory ecosystems and its spread in the European Union, undertaking an original analysis of emerging trends in national sandbox design by sandbox regulators. Section 4 moves to interrogate the dilemma that the regulatory sandbox phenomenon presents for the EU and current and potential future steps towards EU co-ordination on regulatory sandboxes. A defining issue is whether on a cost-benefit analysis EU intervention would produce net welfare gains. Classic arguments against centralisation are weighed against those in favour of intervention in this sphere. The case for the EU to move beyond facilitating stakeholder dialogue to issue guidelines for the operation of regulatory sandboxes in the EU is examined. More radically, the potential achievement of a harmonised EU legal framework for national regulatory sandboxes and the prospect of a cross-border EU sandbox regime are also explored. Finally, informal EU-sanctioned industry sandboxes and pilot regulatory regimes operating on a sectoral basis are characterised as emerging alternative paths of experimental governance. Like the regulatory sandbox, these developments offer an incremental regulatory response to temporary regulatory uncertainty, providing valuable learning opportunities to both institutional and regulatory actors. Section 5 consolidates the analysis, offering concluding thoughts on the prognosis for the future EU regulatory response to the regulatory sandbox pending the delivery of a comprehensive regulatory framework for FinTech.

## The Regulatory Landscape for FinTech in the EU

FinTech innovations have the potential to yield considerable solution-focused benefits as well as delivering efficiencies in time and resources and real economic growth. Transformational changes to financial markets are underway. FinTech is having impacts across all sectors of payments, finance and investments. Existing business models of financial market participants are swiftly shifting and new niches developing. Credit profiling can be carried out using machine learning. Challenger digital banks such as Revolut, N26 and Bunq present competition to bricks and mortar banks. Incumbent banks are also innovating and interested in exploiting the potential of new technologies.^4^ A major development has been the arrival of third party providers (‘TPPs’) such as PayPal and Stripe providing payment initiation and account information services as new services are emerging in the area of internet and mobile payments under the second Payment Services Directive (‘PSD2’).^5^ Furthermore, so-called ‘BigTech’ companies are climbing on board to grab a slice of the FinTech pie, with potential for huge competitive impact given their existing customer bases.^6^ Payments applications including digital wallets such as Apple Pay and Google Pay are expanding. Airbnb, Amazon Pay and Facebook have gained FinTech credentials and authorisations in the EU as e-money institutions. Meanwhile Google is authorised as a payments institution under PSD2 and as an e-money institution and Amazon Lending has its eye on Europe.

Successive technological advances are rapid. The benefits to consumers and society including faster and lower cost distribution of FinTech products and financial inclusion are apparent. Other advances, including those efficiencies expected to be delivered by quantum computing, are awaited. In the insurance arena, InsurTech using big data and AI holds real promise for risk analysis in insurance underwriting.^7^ Equity-based and loan-based crowdfunding are providing alternative sources of capital to traditional funding methods^8^ while the evolving tokenisation trend provides a new investment opportunity for retail and institutional investors. Within the genus of crypto-asset a vast array of products shelter, including cryptocurrencies (such as Bitcoin and Ether) and tokens issued through Initial Coin Offerings (‘ICOs’), offering innovative means of investing and raising capital.^9^ The roll-out of algorithmic robo-advice is changing the face of investment advice. The advent of robo-advice and machine learning driven by algorithms gives rise to new risks including that of incorrect advice and consequent misselling.^10^

Commerce does not, however, occur in a vacuum.^11^ Regulatory burden has a cost and influences forum choice. Currently FinTech innovators seeking to select a location in the EU need to be advised on the potential impact of both domestic laws and EU-driven regulation in each Member State under review alongside other factors such as available labour force, taxation and competition law treatment.^12^ Yet, in the early stages, the reliability of many of the FinTech models being worked upon is uncertain and the risks are not fully capable of being mapped. Accordingly, the novelty that accompanies the new naturally makes both regulators and consumer and investor protection advocates nervous.^13^ A regulatory panacea for many aspects of the FinTech explosion has stumped regulators as technical developments and business models move more rapidly than they can adjust. Furthermore, unenviable regulatory dilemmas exist as economic opportunities and unknowable risks vie with each other for attention.^14^

How long can regulators stand at the side-lines? Risks are often not fully apparent without market access being afforded. A linear pro-competition argument would favour allowing new business models to develop unhindered to aid innovation. Therefore, while technological advances leap forward, it is not surprising that enabling regulatory frameworks for FinTech trail in their wake. FinTech regulatory progress at Member State level is slow and uneven. Risks to investors and consumers may flow from new FinTech models and may not be fully capable of being mapped. Nor are some FinTech activities within extant regulatory scope. Regulatory lag is all too apparent although the position is far from static—the EU is in the early stage of a transitional phase where the regulatory perimeter for FinTech is being clarified. In this phase, rather than been wide-ranging and in force, the development of legal frameworks for the spectrum of FinTech activities is either absent, at a policy discussion stage, or embryonic. This has undeniable commercial and competitive consequences. A legacy regulatory landscape not built with FinTech activities in mind is complex and costly to navigate, sapping the time and resources, particularly of start-ups hoping to find a route to market. Furthermore, a fragmented ‘hit and miss’ regulatory landscape for FinTech across the EU is ripe territory for regulatory arbitrage to play out.^15^ It is opportune for lawyers to play their advisory part as ‘regulatory arbitrageurs’ wading through the waters of regulatory complexities, inconsistencies and silences.^16^

The regulatory dilemma at work is a classic one. To regulate too quickly in the form of reactive regulation risks stifling innovation and hindering it a crucial fledgling stage, yet failing to respond may be to let the FinTech train pass by through inviting geographic displacement or failure of innovation to thrive. FinTech regulation needs to be sophisticated and reasonably capable of standing the test of time, at least in the short to medium term. Alternatively, it needs to be short term and adaptive experimental governance until more mature regulatory strategies can be mapped out. The cutting-edge nature of FinTech innovation means that in most financial systems there is a good deal of uncertainty as to application of regulatory requirements.

Unsurprisingly European Union policy aims to directly work to facilitate Member States to capitalise on the new FinTech era.^17^ A patchy regulatory framework in the internal market across Member States for FinTech creates both regulatory friction and regulatory fragmentation. We are at an early transitional point in the evolution of the regulatory perimeter for FinTech in the EU. Yet to date, in the eyes of some FinTech innovators and Member States, the EU is perceived as moving sluggishly, concentrating on extended stakeholder discussions before commencing a long-drawn out legislative process on the road to adoption of binding instruments and implementation. The Commission’s mid-term review of the Capital Markets Union Action Plan in 2017^18^ squarely recognised the need to realise the potential of FinTech and this and the FinTech Action Plan^19^ prioritised assessing the case for a FinTech licensing and passporting framework for the EU.^20^ Thus far we have two key pieces of bespoke Fintech legislation adopted at EU level. 2020 finally saw the adoption of the Crowdfunding Regulation,^21^ providing an authorisation and passporting regime for equity-based crowdfunding and loan-based crowdfunding platforms. This bespoke framework represented a key deliverable of the FinTech Action Plan, and the first tangible fruits of EU regulatory action albeit restricted in scope to business crowdfunding beneath certain thresholds.^22^ The parallel ancillary Crowdfunding Directive^23^ will exclude authorised Crowdfunding Service Providers from the scope of MiFID II^24^ thus ending uncertainty about its application. Building on this, the Commission’s (2020) Digital Finance Package^25^ exudes ambition to deliver an effective single market for digital financial services based on a passporting model.

The biggest regulatory challenge of all lies in relation to tackling crypto-assets, particularly as they become assimilated into mainstream financial services. Much has been made of the impact of the looming arrival of Facebook’s Libra global stablecoin which heralds a new phase in the evolution of crypto-assets that cannot be ignored. What is undeniable is that asset classes and our society’s understanding of money are changing rapidly.^26^ However, within a single market context the EU is not yet equipped to fully embrace blockchain and existing EU law frameworks stand in the way of establishing secondary markets for securities tokens.^27^ Crypto-assets in all their various forms are still being carefully evaluated and have not yet merited a wholehearted embrace by regulators across the globe, but the approach of many has shifted from hostility to one of cautious curiosity.^28^ As matters stand, crypto-assets of various hues pose taxing challenges for market actors (and regulators) as they seek to understand the legal standing of complex and diverse crypto-assets and how existing regulatory frameworks may impact activities.^29^ One example is the spectrum of regulatory perspectives that exist on ICOs. At one end of the spectrum is outright prohibition—China and Korea have banned ICOs. In the middle, many jurisdictions regulators have chosen to simply issue warnings to potential investors concerning the risks.^30^ At the other end of the spectrum is supervised regulation. Security token offerings take place in France and Germany who have cryptocurrency regulatory regimes. Malta has devised a specific regulatory regime for ICOs.^31^

Tackling crypto-assets as they bed down presents an incredibly compelling challenge for the EU, not least given that the MiFID framework was conceived without the emergence of this genus of asset class in mind.^32^ The Commission has acknowledged the importance of creating a pan-European framework for regulating crypto-assets and has begun to progress legislative proposals. The 2020 Digital Finance Package included a Proposal for a Regulation on Markets in Crypto-Assets (‘MiCA’) that would regulate and enabling passporting in EU capital markets for certain crypto-asset issuers and crypto-asset service providers such as cryptoasset currency exchanges.^33^ In summary, when MiCA comes to fruition, a new dedicated EU-wide regime for issuing and trading crypto-assets will come into play. The focus of MiCA is on providing a bespoke framework to cover crypto-assets that do not currently fall to be classed as financial instruments under MiFID II.^34^ A clear marker is set down to the effect that there is no intention to upend financial market regulation that is already in place; rather the aim is to address what falls outside the regulatory perimeter. Thus crypto-assets that are already classed as financial instruments under MiFID e.g. tokenised bonds and tokenised equities (security tokens),^35^ or as electronic money under the E-Money Directive,^36^ are out of scope of MiCA.^37^ It is planned that MiCA will aid legal certainty by providing an authorisation and regulatory framework for issuing three classes of crypto-asset: (i) asset-referenced tokens (commonly known as ‘stablecoins’); (ii) utility tokens, e.g. Filecoin; and (iii) e-money tokens.^38^ It is significant that stablecoins and crypto-assets service providers will fall within the EU regulatory perimeter for the first time and will be subject to requirements inspired by those applicable to investment firms operating under MiFID II. Imposition of such requirements would assist with ensuring investor protection and market integrity in crypto-exchanges. It should be stressed that this is but the beginning of the negotiation of a complex legislative process that has a projected completion date of 2024. Meanwhile, regulatory lag continues to exert its toll on FinTech.

Although commendable progress is being made, much remains to be done in the FinTech regulatory sphere within the Union. Meanwhile innovation does not stand still. There are many new innovative FinTech applications where it remains necessary to come to grips on a national basis with the MiFID framework and where there are uncertainties of application, disparities in national approach and regulatory gaps. The Commission’s planned work with the European Supervisory Authorities (‘ESAs’) as part of the Digital Finance Strategy^39^ concerning the provision of supervisory and risk-mitigation guidelines around the application of AI-driven FinTech applications provides an example of another much-anticipated future project with an expected delivery date of 2024. Market interest in the potential of data-driven finance is exploding, leading to national regulators being swamped with supervisory queries amid considerable regulatory uncertainty.

At the same time, many efforts at reaching common frameworks and strategies for the single market indirectly cumulatively benefit the FinTech space. The arrival of electronic identification (‘eID’) under the Electronic IDentification, Authentication and Trust Services (‘eIDAS‘) Regulation^40^ enables cross-border digital banking under the Single Digital Gateway Model. The General Data Protection Regulation (‘GDPR’),^41^ PSD2 and the fifth Anti-Money Laundering Directive (‘AML5’)^42^ help to bolster data privacy.^43^ This has particular importance in relation to the increasingly prevalent use of biometric data for authentication purposes in accessing FinTech services. Cybersecurity and digital operational resilience are critically important for FinTech business models.^44^ Meanwhile, the Anti-Money Laundering framework^45^ helps to safeguard the integrity of the financial system but is in need of revision. In addition, a much-needed broader root and branch review of the entire existing EU financial services landscape remain promised but as yet unmaterialised.

Largely inchoate in its realisation, the development of a cohesive EU regulatory strategy for FinTech is disjointed and underdeveloped, rather than comprehensive or complete. What this section has shown is that while worthwhile steps are being taken in the right direction, piecemeal regulatory initiatives cannot individually solve the need for a coherent overall regulatory perimeter for FinTech in one fell swoop. Outdated, inconsistent and incomplete EU financial services frameworks lead to regulatory friction, regulatory fragmentation and uncertainty in FinTech markets, impeding competiveness and cross-border scaling. Forum comparison of widely disparate national regulatory landscapes for FinTech models inevitably fuels regulatory arbitrage. Consequently, the unavoidable conclusion is that even as beneficial emerging elements of bespoke EU FinTech regulation are adopted or on the table, the disenabling effect for innovation continues to apply in the single market. This fortifies the continuing relevance of the unique regulatory adaption in Member States presented by the regulatory sandbox.

## Nature, Design and Spread of National Regulatory Sandboxes

Innovation and its application to existing and new business models moves at a frenetic pace that is not matched by speed of access to market due to the barriers presented by negotiating regulatory complexity, regulatory divergence and regulatory uncertainty. Even though evident progress has been made nationally and supra-nationally, a large gap continues to exist between the inventiveness of state-of-the art innovation in FinTech applications and the inherited creaking and disjointed regulatory landscapes evident at Member State level. The regulatory sandbox phenomenon is worthy of closer attention by policy-makers and regulators given its growing embrace by national regulators.

Characterised elsewhere as ‘opportunity-based regulation’,^46^ the regulatory sandbox deserves to be lauded as an inspired feat of regulatory inventiveness, providing holding space for disruptive technologically-driven innovation to be tested at a micro level under the benign watch of a financial regulator. In the absence of a fully articulated proportional regulatory framework for FinTech in its many forms, the regulatory sandbox concept fulfils a valuable gap-filling role that is mutually beneficial for both regulatory subjects and regulators. It is a solution-focused, glass half full mechanism. Rather than focusing on the ‘problem’ presented by the frustrating inadequacy of legal frameworks when presented with new technological interfaces, the focus of the regulatory sandbox is instead on recognising innovation potential and facilitating an understanding of the labyrinthine regulatory maze with a view to realising consumer benefit. Regulators who provide a regulatory sandbox are responding to an international scramble for FinTech market share and are adapting and innovating in terms of their sandbox offerings in a bid to attract business, recognising that a regulatory sandbox can open a welcoming door to innovators contemplating where to locate. Simply put, participation in a regulatory sandbox assists tech innovators. First, a regulatory sandbox helps them to negotiate the rocky waters of successfully testing an experimental product or service in a controlled testing environment with monitoring from the regulator before bringing it to a full-scale market launch.^47^ Secondly, the sandbox environment often offers precious, hands-on regulatory assistance to innovators navigating the uncertain application of a financial system’s complex web of legal rules not designed with the FinTech innovation in question in mind. The regulatory sandbox is thus designed to help get novel technological products and services over the line and into the market and small scale testing minimises the costs and risks of failure. This is a game-changer for FinTech innovators, particularly start-ups with limited funds at their disposal.

In terms of the origination of the regulatory sandbox, the United Kingdom’s Financial Conduct Authority (‘FCA’) can claim the credit for being the regulatory trailblazer, pioneering the regulatory sandbox for FinTech with a view to counteracting regulatory lag and complexity as part of its Project Innovate. The FCA’s regulatory sandbox was launched in 2016 and has since been widely emulated by regulators all over the globe.^48^ In the period from the emergence of the first regulatory sandbox model pioneered by the FCA, regulatory sandboxes have multiplied across regulatory landscapes in both developed and developing countries.^49^ Within the EU innovation hubs/facilitators offering support and regulatory advice to FinTech innovators have sprung up across much of the EU and are the predominant mode of support offered by EU regulators.^50^ Indeed, although as discussed below there is clear readiness to embrace the more radical sandbox option, there also exists a competing perspective among some regulators and observers that the regulatory sandbox’s cousin, the innovation hub, represents a less costly way to help a greater number of FinTech innovators to get to grips with the regulatory architecture, while also allowing some regulators the opportunity to learn about new business models and technological advances.^51^

Within the EU, roll-out of the more radical regulatory sandbox as a bespoke regulatory offering is growing steadily year on year with eight (or almost a third) of the 27 Member States offering regulatory sandboxes or having put in place legal rules to establish them. Regulatory sandboxes are in operation or on the way pursuant to national legislative frameworks in Austria,^52^ Denmark,^53^ Hungary,^54^ Latvia,^55^ Lithuania,^56^ Malta,^57^ the Netherlands,^58^ and Spain.^59^ Regulatory responses to FinTech are influenced by observation of other regulators’ actions and, as matters stand, regulators in a further five Member States are committed to following suit, many with advanced preparations underway to establish a regulatory sandbox. Estonia, which has a well-developed technology sector, and a pro-start-up culture that includes a favourable tax environment, is in the process of establishing a regulatory sandbox in conjunction with the European Banking Institution for Development and Reconstruction, as are Greece and Poland.^60^ Bulgaria and Italy are also committed to launching regulatory sandboxes. This holds the prospect of a sizeable 48 percent of EU Member States hosting a regulatory sandbox regime in 2021. Following a 2020 public consultation, a further Member State, Slovakia, is also considering whether to launch a regulatory sandbox. Other EU regulators may decide to follow suit. Meanwhile, within the wider European competitive landscape for FinTech, in addition to the United Kingdom, Norway and Switzerland have regulatory sandboxes. Consequently, although it is early days for the regulatory sandbox, as a phenomenon that only germinated in 2016, this regulatory stopgap has gained a considerable foothold within the regulatory topography and FinTech framework of EU Member States and in jurisdictions with whom they vie for FinTech space.

The establishment of a regulatory sandbox is an emblem of a potentially FinTech-friendly jurisdiction. All other things being equal, EU-based sandbox regulators and their jurisdictions are likely to benefit from a competitive bounce in FinTech business from FinTech operators looking for an EU host State. Sandbox regulators hope to also benefit from the technological and regulatory learning opportunities provided by hands-on monitoring at close quarters provided to sandbox participants. The dynamism and iterative learning places the regulatory sandbox within the realm of experimental governance architecture.^61^ Close monitoring provides an opportunity for regulators to gain an understanding of innovation, potential risk to consumers, and how an existing regulatory framework may apply^62^ as well as allowing gaps in the regulatory and supervisory framework to be identified. Monitoring reduces risk, while enabling mutual learning, helps ensure the best possible outcome and can improve speed to market. As has been pointed out by a European expert group, ‘[i]f the business activities are not yet regulated, but might in future become a regulated activity, the sandbox participant and the sandbox program should help inform an assessment of whether or not this business needs to be regulated’.^63^ There are already some early movers. For example, in 2019 the Danish financial services authority used the knowledge gained from its regulatory sandbox to formulate recommendations for financial institutions concerning the use of supervised machine learning. It should nonetheless be borne in mind that the potential for regulatory learning is somewhat restricted by the very small numbers admitted to a regulatory sandbox and also to nature of the business models that come though. It is usual for the size of the cohort accepted into a sandbox round to be kept fairly small.^64^ For example, Denmark’s FT Lab is limited to five participants at a time. This does somewhat reduce a regulator’s line of sight. Indeed, the question has been raised of whether ‘an applicant-driven sandbox is the most effective way to collect and curate [regulatory] insights’.^65^

Regulatory sandboxes are not all plain sailing. Considerable resources are needed for the intensive monitoring required of regulators during the testing phase. Furthermore, adoption of the regulatory sandbox model can only do so much—it should not be mistaken for a panacea overcoming all regulatory shortcomings. While the provision of a regulatory sandbox by a regulator may purport to indicate an openness to FinTech innovation, careful scrutiny is needed by individual market actors as to a given sandbox regime’s actual cost-benefits within the sandbox environment and, even more crucially, upon exit. Well-established incumbent market players and BigTech companies, who are well-resourced and well-advised, with a strong market recognition factor and existing client base, may not need the leg up a regulatory sandbox offers even if they are increasingly moving into territory traditionally associated with financial institutions.^66^

Lack of uniform, transparent information on eligibility, admission and outcomes in relation to regulatory sandbox regimes severely inhibit the ability to make sophisticated cross-jurisdictional comparisons concerning how regulatory sandboxes already in operation are faring. Nonetheless, given the foothold gained by regulatory sandboxes within the EU, and the burgeoning interest displayed by EU institutions, it is opportune to reflect generally upon design choices facing regulators in the design and operation of regulatory sandboxes. A comparison of publicly available information concerning the various types of sandboxes on offer reveals both relatively standard and competing design choices at play. To set the stage for discussion of an EU response to the regulatory sandbox, some key aspects of sandbox design are examined below, before moving in Sect. 4 to consider the potential for a co-ordinated EU response.

### Threshold Eligibility Parameters for Regulatory Sandboxes

A pivotal threshold issue for regulators concerns setting parameters around sandbox eligibility. Consistent with the controlled hothouse environment of a regulatory sandbox, each regulator operating a regulatory sandbox makes threshold choices in relation to regulatory appetite centring on the types of activities and market players that it will comfortably admit in principle to the sandbox environment. The profile of candidates eligible for regulatory sandboxes is usually set to be broad. This allows established players such as incumbent banks as well as start-up entities to be considered. Furthermore, a broad applicant profile potentially leads to a breadth of applicants and combinations, mirroring market supply-side demand. In terms of applicants, there is a trend for technology companies to partner with established financial institutions.^67^ Some regulatory sandboxes are specialist, restricted to a defined segment of the market. For example, Bulgaria is establishing a RegTech sandbox to be known as ‘Sofia RegTech’. Hong Kong has an InsurTech regulatory sandbox. Most regulatory sandboxes in the EU (and outside) are constructed to cover a broad range of FinTech applications. There is fierce competition for the FinTech market and most regulators are aware that an unduly restricted approach to eligibility may doom their regulatory sandbox by generating poor levels of early stage interest and/or poor conversion to actual sandbox applications.

In setting the scope of qualifying activities of a regulatory sandbox, there are sizeable challenges for regulators when met with new technologies and business models in considering the important threshold question of whether certain activities ought to be excluded from eligibility for a regulatory sandbox on policy grounds. Clearly a balance is needed between not trampling on invention and not allowing risks to develop unhindered. In the fast-moving and nuanced world of FinTech investment, establishing that set point and justifying it from a regulatory perspective is a far from straight-forward task.

Before the emergence of the MiCA proposal, and while it continues to remains simply a proposal, crypto-assets have presented national sandbox regulators with a real conundrum as they offer a Gordian knot of opportunities and risks that are not fully mapped or regulated. Inherent technological vulnerabilities are also a matter of concern. Some jurisdictions have been more conservative when framing sandbox parameters. For example, the regulatory sandbox operated by the Reserve Bank of India excludes ICOs and cryptocurrencies/crypto-asset services.^68^ Prudence is justifiable. Nonetheless, static regulatory stances around sandbox scope may prove too rigid as markets adapt and move, hence the importance of regularly reviewing scope. That sensibility is already in evidence in EU sandbox jurisdictions. The regulatory sandboxes in existence in Denmark, Hungary, Lithuania, Poland, and the Netherlands do not provide blanket exclusions; rather each operates an ‘open arms’ policy, welcoming innovation and relying on applying risks and benefits assessment criteria to determine suitability of applications. This reflects the fact that the financial services industry is gradually becoming accustomed to transformational change and assimilating it. For instance, a gradual level of comfort is being attained in place of scepticism in relation to blockchain’s beneficial potential to transform capital markets, improving access to and cost of capital in the process. There is a strong argument to be made that allowing some crypto-asset activities to potentially come within a sandbox is valuable precisely because controlled experimentation in such an environment allows insights to be gained by regulators about risks and vulnerabilities alongside potential consumer benefits. There are examples of national sandbox regulators who have embraced this perspective. The UK’s FCA, the most seasoned administrator of a regulatory sandbox, has taken the plunge of admitting participants testing crypto-asset-related propositions into its sandbox cohort.^69^ Russia’s central bank has admitted a project for stablecoin linked to real property to its regulatory sandbox. Some regulators have specifically targeted crypto-asset sectors. The Reserve Bank of South Africa issued a specific call for sandbox applications from firms working with blockchain and cryptocurrencies. Brazil decided to specifically adapt its regulatory sandbox rules to take account of developments in this sector. This shows a level of willingness among sandbox regulators to engage and learn.

### Selection Criteria for Admission to National Regulatory Sandboxes

Selection criteria provided for national regulatory sandboxes are often indicative rather than exhaustive. As such, a measure of discretion applies to regulatory decision-making; this inevitably calls for some *ad hoc* thinking by regulators reviewing applications on a case by case basis. Pre-formulated selection criteria applied by sandbox regulators to applications for admission to the sandbox determining who will qualify for admission to a particular sandbox are generally heavily influenced by the UK prototype. Reflecting the original FCA model, for most sandboxes these tend to focus primarily on the twin peaks of (i) innovation and (ii) market benefits in the form of efficiencies or bringing new services to market. However, the combination of innovation and potential consumer benefit should not, of itself, ensure a free pass into the welcoming atmosphere of a sandbox. A realistic assessment is also needed of what is likely to be substantively gained by the prospective sandbox user from time-limited exposure to the cosseted sandbox environment. Building on the UK’s pioneering approach, it is usual to specify that there must be a justified need to test the relevant innovation within a regulatory sandbox environment. This may be satisfied by highlighting the difficulties that present in fitting what is proposed into the existing regulatory framework. Notably, Australia’s 2020 reworked Enhanced Regulatory Sandbox regime operated by the Australian Securities and Investment Commission added a net public benefit test to the mix. Therefore not only must there be innovation, the proposed activity must be likely to create public benefit that outweighs any possible public detriment.^70^ Furthermore, deriving from the UK model, it is usual for the guidelines to specify that to be eligible for admission an innovation must be at an appropriate stage for testing.^71^ Readiness to test relates to a number of matters, not just those relating to product development, for example, a business plan, competent staff and management, appropriate governance structures, potential clients for the test. Finally, there ought to be a particular benefit to be gained from experimental controlled live testing (other than simply access to free regulatory advice).

### Time to Decision on Sandbox Applications

Time to decision has emerged as an important constituent element of the pro-FinTech innovation approach being broadcast by sandbox regulators. Globally, many sandbox regulators accept applications on a cohort basis by announced round (as seen within the EU in Denmark). An alternative is to accept sandbox applications on a rolling basis. Rolling applications are a feature of FinTech sandboxes in Austria, Latvia, Lithuania, Malta, and the Netherlands. In some Member States (such as Denmark, Latvia, Malta and the Netherlands) sandbox regulators provide no guarantees as to how long applications will take to process. Hungary’s MNB (Central Bank) permits itself up to three months to decision,^72^ while in Austria the FMA commits to a decision within six months. The Bank of Lithuania takes one to two months to review applications and another one to two months to collaboratively agree the testing conditions. Use of a block exemption licence model, as seen in Australia, ingeniously removes the need for application assessment and notification of a decision.^73^ Australia’s block exemption or white list approach allows firms to satisfy themselves that they come within criteria for validation testing and to notify of an intention to test without any requirement of an approval being issued.

Where a guarantee is provided as to time to a decision this can be distinctly relevant to the overall deliberations of a would-be regulatory sandbox applicant in deciding to which regulatory sandboxe(s) to apply. Time to decision has become akin to distinctive plumage—a competitive calling card for some regulatory sandboxes and their countries in a bid to attract FinTech players. Outside the EU, express decision-making has begun to feature competitively as sandbox operators have sought to sharpen their inter-jurisdictional competitive advantage in a crowded global marketplace. This is done by sandbox regulators committing to a highly truncated time to a decision on whether applications to be admitted to the sandbox are successful. Consistent with Asia’s pre-eminence in FinTech, the truncated decision-making model originated there. The Central Bank of Malaysia is committed to 15 working days in determining applications to its FinTech sandbox^74^ while Singapore’s Sandbox Express has a 21 day model applicable to particular pre-defined sandboxes and certain business areas.^75^

Fast-tracked decision-making raises concerns as to the potential to compromise appropriate fitness and propriety assessment, as well as compromising risk assessment.^76^ This concern particularly arises for those regulators who are not appropriately resourced or as well-versed in technological innovation as regulators in jurisdictions of choice for FinTech innovation. All in all, any evidence of expedited decision-making emerging within regulatory sandboxes operating in the EU should be a matter of concern in relation to standard-setting within the EU. Decisions taken by regulators on sandbox admissions need to be suitably informed based on an appropriate review of relevant matters concerning the proposed projects and their protagonists so as to avoid unleashing risk. No fixed time limit should be permitted to impair this.

### Sandbox Duration

Within those EU Member States offering regulatory sandboxes, the shortest defined testing period offered is three months (Latvia) followed by six months (Denmark, Latvia, Lithuania and Malta) and 12 months (Malta).^77^ Globally, up to 12 months is a commonly defined sandbox period.^78^ Some jurisdictions (including Austria) offer a longer sandbox experience of up to 24 months.^79^ An alternative approach is to define the duration of the sandbox on a case by case basis as occurs in the Netherlands.^80^ Clearly the period of the sandbox varies across sandbox design. National divergences may be of less significance in practice given that regulatory sandbox schemes generally specifically envisage that the initially defined period can be extended by mutual agreement.^81^ Extensions tend to be restricted to cases where initial testing has proved promising and extended testing appears beneficial in order to address specific issues that manifest during the initial test bed. An alternative is to deploy a size-based criterion to determine when the sandbox must be exited as seen in Switzerland.

### Addressing Risk during Sandbox Testing

As the EU institutions consider how to respond to the proliferation of the regulatory sandbox, the most vital considerations are those in relation to risk mitigation. Currently each sandbox operator in the EU autonomously determines how best to mitigate risks associated with controlled FinTech experimentation in a sandbox environment. The crux is that many of these risks are unknowable prior to testing. In assessing sandbox applicants, Denmark’s FT Lab highlights the need for the applicant to have ‘access to necessary competencies and resources to lead the project prudently’.^82^ Given the gatekeeper function of regulators, competent personnel and adequate resourcing are fundamental. Hand in hand with sandbox participation lies a corresponding intensive monitoring obligation for sandbox regulators while they guide sandbox participants through what can be an intransigent and complex regulatory thicket of domestic and EU-derived financial services rules.

The risk of loss to clients in the testing period and provision of suitable redress mechanisms needs to be front and centre in any sandbox regulator’s mind, no matter how FinTech-friendly an image they project. Although testing involves controlled small-scale experimentation, it is necessary for sandbox regulators to consider potential risks to retail and institutional investors and the potential for systemic risk, and to tailor-make an approach for each sandbox participant. Restrictions imposed will usually seek to contain the scale and capital involved. There may also be restrictions on the number and type of consumers and transaction values. In Poland, the KNF sandbox does not allow sandbox participants to collect funds from clients. A financial compensation solution may be imposed. An alternative is to permit insurance cover to provide redress in the form of client compensation for loss (as seen in Hungary).

Risks such as privacy, fraud and cyber-threats also need to be taken seriously even during scaled-down testing in a regulatory sandbox. Reflecting the risks involved for clients of a FinTech firm within a sandbox beta-testing operation, it is standard practice for sandbox participants to be required to make specific disclosures to clients during testing to warn them of risks and to advise of dispute resolution and redress options. For example, participants in Denmark’s FT Lab must use specific wording to advise clients during the testing phase of the risks and that participation does not constitute an endorsement of the product by the regulator.^83^

### Regulatory Flexibility

While beckoning to FinTech innovators, sandbox regulators within the European Union cannot simply jettison the EU financial services rulebook at will for sandbox testing. There is, however, some limited in-built flexibility available to EU sandbox regulators in relation to the regulatory perimeter. A number of distinct scenarios arise for EU regulators devising and implementing a sandbox regime. In any situation where there has been maximum harmonisation of relevant legal rules (as applies under PSD2), there is little room for manoeuvre. However, some legislation at EU level such as CRD IV^84^ and PSD2 has an in-built proportionality aspect. This may provide Member States with some wriggle-room for small-scale sandbox experimentation. On the other hand, some Member States have chosen to gold-plate EU legislative instruments with additional requirements. For policies adopted by the ESAs, these generally afford a measure of freedom to interpret them flexibly provided it can be shown that the legal and regulatory aims are satisfied by alternative means.^85^ Where national rules permit exceptions or flexible interpretation, there may be scope for regulatory relaxation and the provision of a tailor-made sandbox for individual participants. At a domestic level, policies and standards of national supervisory authorities which fall short of legislative rules offer most scope for adaptation. Furthermore, differing bespoke conditions may be imposed on individual sandbox participants by sandbox regulators across the EU.

This gives a sense of the vast array of different possible permutations of regulatory treatment in a free for all competitive sandbox environment that is not subject to EU harmonised rules or guidelines. The fragmented regulatory landscape for FinTech and the absence of an EU approach to regulatory sandboxes means that it is clearly not a case of uniform rules applying to sandbox participants no matter which territory they choose to test in within the EU.

### Reporting Requirements

Reporting requirements are an important source of feedback for sandbox regulators. For that reason, periodic reporting should be required during testing. For reflective learning, it is also important for sandbox participants to be required to provide feedback on the ultimate outcomes of the sandbox after exiting. A good example of this is evident in Malaysia where sandbox participants must report to the Bank Negara Malaysia within 30 days of exit on key outcomes, measuring the success or failure of the test against key performance indicators and lessons learned from testing.^86^ They are also required to notify the regulator of incidents during testing and to provide information on the resolution of customer complaints.

### Sandbox Exit Strategy

Whatever the outcome of the controlled testing phase, a well-managed sandbox exit is critical. This counts as a key aspect of successful sandbox design which must be meticulously thought through. Sandbox participants need to be made aware of the importance of devising an exit strategy with a view to appropriately transitioning to independent market operator with a roll-out of services on the open market. There is also the very real prospect that the testing phase does not produce a viable outcome. Other than a successful transition to market, most commonly, a sandbox arrangement will be terminated where it becomes clear that the testing is not capable of viably realising the hoped-for outcomes. In this instance the requirement to have a credible exit plan is an important consumer protection measure. The exit pathway should build in mechanisms to address outstanding obligations to clients.

Finally, a well-planned regulatory sandbox framework ought to build in leeway for termination by the regulator in other circumstances. Thus, where a sandbox participant breaches a fundamental condition of sandbox participation, the relevant regulator should be able to activate a contractual right to revoke approval for continued sandbox participation. The type of fundamental conditions that could be specified as triggering a right to terminate sandbox participation could typically relate to matters such as material inaccuracies in the sandbox application, data security breaches, fraud and repeated service disruption. It would also be prudent to specify that exit may also be prematurely forced under the terms of a sandbox arrangement where a flaw emerges in a FinTech product that could adversely affect consumers or the financial system. This could occur for example, if it emerged that a robo-advice product being tested had been incorrectly programmed leading to incorrect investment advice being provided.

Having considered the spread within Member States of the regulatory sandbox and key design features of the regulatory sandbox, the next section evaluates how the EU has responded to date and the options for how it may act in the future.

## Steps Towards EU Co-Ordination

Looking first to classic arguments that could potentially be marshalled against a centralised EU approach, a leading perspective from the economics literature is that there needs to be accommodation for when centralisation does not add up, seen most obviously in the emphasis in the post-Maastricht Treaty era on subsidiarity.^87^ The subsidiarity principle recognises that sometimes Member State autonomy and regulatory competition between Member States may yield better outcomes over EU intervention aimed at a common approach. Taxation regimes, laws and regulation and economic policies influence location choice for otherwise mobile agents. In this regard governments can be seen as being actors in institutional competition with each other in relation to a market for mobile freedom of establishment. In principle competition between Member States and regulatory systems may exert incentives on governments to increase competiveness. Market failure may not occur if a small number of influential jurisdictions adopt a meaningful regulatory approach that leads others to come along.^88^ However, context is everything and institutional competition among Member States does not inevitably lead to efficient outcomes, in which case supplementary EU intervention may be warranted to redress adverse outcomes associated with partitioning along national lines.

Having regard to the burgeoning spread of the regulatory sandbox in Europe and globally, a disparity of approaches to sandbox operation, and a lack of transparency are presenting as problematic to the single market concept upon which the European Union is founded. Some Member States passively await potential EU action rather than acting strategically to articulate a developed regulatory framework for either FinTech or regulatory sandboxes. In such cases the focus may instead be on wooing potential FinTech inward investment through portraying a ‘FinTech friendly’ national environment. EU scholars have rightly warned of the unpredictable outcomes associated with relying on regulatory competition as necessarily superior to harmonisation.^89^ It may not be sufficient to rely on Member States to come up trumps. Leaving regulatory sandboxes to institutional competition may lead to sub-optimal regulatory consequences under the problem of ‘zero-regulation’.^90^ In the present context where capital is highly mobile, the potential for a proverbial race to the bottom in the Member States in a bid to attract FinTech inward investment is unfortunately real.^91^ In other instances a market may remain under-developed. Indeed, this rationale has partly underpinned the Commission’s DLT Pilot Proposal.^92^

A defining issue then is whether on a cost-benefit analysis regulatory harmonisation would produce net welfare gains. The likely benefits of the EU institutions playing a leading role in broadly co-ordinating regulatory approaches is particularly apparent in the FinTech space. Regulation of new technologies represents a highly complex task for national regulators. Moreover, business models can be delivered virtually making borders opaque. Given the potential value contribution of FinTech to national economies, leaving matters to institutional Member State competition is replete with the potential for regulatory arbitrage. National regulatory fragmentation and a race to the bottom is almost inevitable. Furthermore, national responses cannot offer a viable alternative to an EU cross-border passporting solution for services provided based on home state authorisation—that can only be achieved by supra-national regulation. In short, as acknowledged in the Digital Finance Strategy,^93^ where cross-border trading is the aim, fragmented Member State approaches force resource intensive compliance costs on putative market entrants.

Accordingly, within European Union policy, there is a well-leavened realisation of the need to support FinTech innovation and harness a digital single market, as enshrined in the FinTech Action Plan.^94^ A competitive, ‘frictionless single market’ in the digital sphere is part of the Commission’s five year digital strategy.^95^ That ambitious goal will take time and effort to deliver on. In the meantime, enhancing cooperation and dialogue among regulators on how to facilitate FinTech in a co-ordinated manner holds the prospect of easing the path for innovators in the EU even in the absence of agreed common regulatory approaches that would provide a level playing field for FinTech activities. Stakeholder dialogue is a well-established *modus operandi* within the European Union; before the EU institutions act, they listen and reflect. Stakeholder engagement is fundamental to this. Innovation hubs and regulatory sandboxes across the EU are providing a vital point of contact and engagement between regulators and FinTech entrepreneurs of all hues. The feedback gained in relation to regulatory hurdles and gaps can inform cross-EU inter-regulator dialogue. Informal dialogue and information gathering from EU regulators is particularly important given the opportunities and risks that new technologies and business models pose. Information-gathering at EU institutional level also takes the form of reports by the ESAs in relation to regulatory issues and national responses to innovation in the form of innovation hubs and regulatory sandboxes.^96^

But is information-gathering and sharing among stakeholders enough or could more concrete steps to be taken by the EU? Based on information gathered to date at EU level, the way forward for EU policy concerning regulatory sandboxes provides the EU institutions with a classic regulatory dilemma involving policy choices with incremental stepped power effects. It must choose between between (i) continuing to simply facilitate stakeholder dialogue; (ii) issuing soft recommendations or guidelines for operation of national regulatory sandboxes, through to (iii) full harmonisation of rules on sandboxes. These policy choices are explored below and the prospects for establishment of an EU cross-border regulatory sandbox regime are evaluated. The analysis concludes with an assessment of some current evidence showing that the EU is favourably disposed towards embracing some forms of sectoral experimental governance.

### Facilitating Stakeholder Dialogue

At the base of the pyramid of EU regulatory options concerning the emergence of the regulatory sandbox phenomenon within the EU lies that of stakeholder dialogue given its informal nature focusing on peer to peer interactions and relationships. Inestimable practical benefits accrue from stakeholder dialogue based on the benefits of relationships created and maintained and the learning value from the information shared. Stakeholder discussion represented a central plank of the European Commission’s FinTech Action Plan emanating from DG FISMA.^97^ The joint report in 2019 from the ESAs on innovation facilitators^98^ also emphasised the importance of increasing co-operation.

Informal infrastructure is in place to encourage regulatory dialogue and encourage FinTech innovation within the EU. Two significant initiatives allow EU regulators to upskill in relation to FinTech by enabling material enhancement of their understanding of how FinTech applications work and are impacted by regulation. First, the EU FinTech Lab has acted as a conduit since 2018 for sharing information and training between European and national regulators and supervisors. It also provides a forum for them to meet with and learn from technological innovators. Secondly, in response to Member States responsiveness to facilitating FinTech innovators bringing their product to market, 2019 saw the launch by the Commission of the European Forum for Innovation Facilitators (‘EFIF’).^99^ This network facilitates dialogue among supervisors and encourages discussion on common approaches within the EU and with third countries on the regulatory treatment of technological innovation.^100^ It has representation from each innovation hub and regulatory sandbox in Member States and the ESAs are also represented. The European Banking Authority (‘EBA’) also has a FinTech Knowledge Hub for regulators. To be clear, no legal effects can result. Any common positions arrived at cannot be enforced; they have no standing, and fall below soft law. Nonetheless, dialogue in these fora may usefully coalesce views on best practice approaches to the operation of regulatory sandboxes in the EU, and as to how regulation may evolve to fit FinTech.^101^ This is the essence of the spirit of the new governance approach to regulation which has been well dissected in the literature on EU regulation.^102^

Given the lack of real clout associated with dialogue, it is unsurprising that the ESAs envisaged that the establishment of an EU network of innovation facilitators could act as a useful knowledge-sharing forum, but only as part of a ‘multi-pronged approach’.^103^ In the FinTech Action Plan, the Commission gave credence to this, and as a preliminary step the ESAs were tasked with charting authorisation and licensing approaches as applied to FinTech business models across the Member States and with exploring the vexed question of how national authorities approached questions of proportionality and the application of flexibility.^104^ To conclude, while continuing stakeholder dialogue around the operation of regulatory sandboxes is valuable, more action is needed to develop a new governance approach founded upon stakeholder participation that ultimately leads to a bottom up framework.

### Issuing Non-binding Guidelines for Regulatory Sandboxes in the EU

At the second level of the EU regulatory pyramid lies the option of standard-setting for regulatory sandboxes in the EU. This could take the form of a European Commission recommendation creating non-binding guidelines for Member States.^105^ Indeed, the European Commission committed to providing a blueprint for national sandboxes in the form of best practice guidelines on regulatory sandboxes in its FinTech Action Plan^106^ but has not yet delivered on this ambition. While hard law instruments require more precision and a lengthy legislative gestation, non-binding guidelines with EU imprimatur can have the advantage of more flexible drafting and swiftness in terms of adoption and revision. Furthermore, they fit within the post-Lisbon ‘open method of co-ordination’ involving co-ordination at an intermediate level between regulatory competition and harmonisation.^107^ This has particular resonance for FinTech financial markets where market definitions and business methods are in flux. Indeed, the issuing of non-binding guidelines has become a defining *modus operandi* in the field of financial supervision since the establishment of the ESAs.^108^

The Securities and Markets Stakeholder Group suggested that some level of co-ordination spearheaded by the European Securities and Markets Authority (‘ESMA’) would help with both quality and transparency.^109^ Following on the foundations laid by the work that has been jointly done by the ESAs on mapping national approaches and in identifying issues that need to be tackled,^110^ the Commission’s report on best practices for regulatory sandboxes is awaited. Furthermore, the Council has requested the Commission to study regulatory sandboxes and the use of experimentation clauses allowing regulators a measure of flexibility in relation to the application and of legal rules to the testing innovation within a regulatory sandbox.^111^

Questions of competence loom large when possibilities of the ESAs adopting new guidelines or recommendations are mooted.^112^ Do the ESAs possess the requisite competence to step in? The Commission’s 2014 Report on the ESAs highlighted that guidelines and recommendations would have to meet the cumulative criteria of (i) establishing ‘consistent, efficient and effective supervisory practices’ and (ii) ensuring the ‘common, uniform and consistent application of Union law’.^113^ The continuing value of this function was validated by the Commission in the Digital Finance Strategy.^114^ As decentralised agencies rather than EU institutions, it could be considered challenging to identify a legal basis for the ESAs to be tasked with producing guidelines on regulatory sandboxes where there is no underlying EU legal framework in the field. However, the ESAs’ role has evolved in recognition of their contribution. An expanded remit deriving from Article 9(2) which was inserted into the ESA founding Regulations with effect from 1 January 2020^115^ gives the ESAs the obligation to ‘monitor new and existing financial activities’ and the power to adopt guidelines and recommendations ‘with a view to promoting the safety and soundness of markets, and convergence and effectiveness of regulatory and supervisory practices’. On its face this Article 9(2) power would permit the ESAs to issue guidelines on regulatory sandboxes that were predicated upon ensuring investor protection and financial stability.

Irrespective of which body or bodies are ultimately tasked with setting and owning any resulting guidelines, the issues of content remain the same. What should such guidance on operating regulatory sandboxes in the EU cover? Leaving to one side the rather intractable issue of available regulatory flexibility with regard to the application of substantive legal rules to sandbox participants, it is not difficult to pinpoint key matters upon which uniformity would be desirable. A broad principle of equality of access to regulatory sandboxes by start-ups and incumbents would be a desirable starting point. This principle was emphasised by the Commission’s Expert Group on Regulatory Obstacles to Financial Innovation in stating that ‘[a]ll market participants should be treated equally: irrespective of the size or degree of establishment on the market, innovators of all kinds should be able to apply without discrimination’.^116^ Transparent and objective criteria regarding eligibility for admission to a regulatory sandbox would underpin this.

In some Member States the selection criteria used by sandbox regulators may not qualify as sufficiently transparent. Lack of transparency is compounded by the unique nature of the regulatory sandbox such that often the relevant criteria are not set down in legislation or formally adopted rules. However, lack of formally set out rules can lead to unfortunate uncertainty among regulatory actors on relevant sandbox criteria. It may also cast doubt on the objective nature of relevant rules and their application in decision-making on sandbox admission. This may be contributed to by the informal communication style adopted by some regulators who are keen to convey above all else that they are ‘open for FinTech business’. Thus regulators’ websites may encourage contact from FinTech entrepreneurs and provide little or no detail concerning sandbox terms and conditions online before exploratory dialogue in relation to regulatory sandbox entry takes place with a potential applicant.^117^ Consequently, for the pre-application stage, EU guidelines could usefully set transparency expectations surrounding the type of minimum information to be publicly provided on the websites of EU regulators offering regulatory sandboxes.

Outside the EU, there are instances where there is a trade-off between transparency and an underlying preference of some regulators for a direct approach to be made to them by the regulatory actor so as to initiate regulatory dialogue. An example of this is seen in Hong Kong where fully transparent parameters for relevant Securities and Futures Commission Sandbox^118^ and the Insurtech Sandbox^119^ are not provided online. Instead regulatory dialogue is encouraged to determine operation and fit. An example of a more radically transparent approach is that provided by the shared Canadian Securities Administrators’ (‘CSA’) Regulatory Sandbox. Rather than providing a set of admission guidelines, the CSA provides a strong level of transparency to regulatory actors through providing online public access to previous decisions including the terms and conditions imposed on sandbox users.^120^ This reflects the fact that the CSA model is individualised, based on providing tailored exemptive relief from securities laws requirements.^121^

Agreement on fundamental standards of best practice for regulatory sandboxes could help to avoid FinTech regulators being drawn into a race to the bottom in terms of admission criteria and post-admission conditions attached to sandbox participation. In the quest for a common EU approach to assessment criteria for admission to regulatory sandboxes some consensus can be discerned across sandboxes and readily distilled into guidance on relevant factors in assessing the applications for sandbox testing of new business models and technological innovation. These include matters such as need and readiness for testing in a sandbox environment, innovation in the sense of novelty or novel application, benefits to consumers, for example, through providing quicker and more cost-effective options. On the flip side, risks to consumers need to be carefully weighed in the balance and may negative potential benefits. Important issues to be treated concern investor protection measures, public transparency and regular reporting on the sandbox experience in practice. It may make sense for there to also be overall reporting by national sandbox regulators of outcomes to ESMA. Such guidelines could also add some precision in relation to disclosures to consumers and the type of risk-mitigation measures that could be imposed. Recommended exit protocols could be formalised to cover both situations where controlled exit does not lead to full product roll-out as well as best case outcomes where successful testing leads to anticipation of scaling up through a full market launch.

The ESAs have highlighted a very real issue within the context of innovation hubs (and the point applies even more to regulatory sandbox participants)—that firms could mistakenly rely on general indicative guidance provided as having a final, legally binding quality.^122^ Given the regulatory complexities at work, a sandbox regulator offering assistance in a test bed should take care not to appear to replace the need for specific legal advice concerning all aspects of product roll-out (including those aspects outside of financial services such as data privacy). An expectation in such guidelines that clarifying wording on this should be prominently on the relevant regulator’s regulatory sandbox webpage and in relevant documentation would be sensible. In addition, it would be prudent to require careful wording to be employed so as not to give the mistaken impression that a regulator operating a regulatory sandbox is in some way endorsing either the sandbox applicant or the proposition being tested.^123^

The Commission in the Digital Finance Strategy^124^ has adverted to a commitment to respect the principle ‘same activity, same risk, same rules’, so as to create a level playing field between existing financial institutions and new market entrants. In line with that the ESAs firmly state the position that regulatory sandboxes cannot allow EU regulatory requirements to be relaxed, but that available proportionality levers may be applied.^125^ This stumbles upon the problem of the uneven EU financial services playing field at national level, the effects of which FinTech players and their lawyers are not equipped to assess other than through a painstaking process of individual regulatory dialogue with national regulators and supervisors. This brings us neatly to consideration of the complex question of whether the EU might be prompted to target some form of harmonised legal framework for regulatory sandboxes.

### A Harmonised EU Legal Framework for Sandboxes?

A more radical approach for the EU institutions to the regulatory sandbox explosion amid unanswered regulatory challenges for FinTech would be to create a harmonised legal framework for the operation of regulatory sandboxes in the EU. The main argument for this is the classic internal market one: that removing cross-border dissonance would assist with the objectives of the single market and the realisation of the potential for scaling up of FinTech more or less on a level playing field across multiple markets. In relation to the content of any such common framework, looked at in the abstract, much of the discussion above in Sect. 4.2 concerning the potential content of non-binding guidelines on the operation of regulatory sandboxes, could equally be formalised into an EU harmonised framework for regulatory sandboxes. Policy nudges in the direction of the pursuit of a harmonised approach were cemented in the report of the European Commission’s Expert Group on Regulatory Obstacles to Financial Innovation which came out strongly in favour of harmonising standards across the EU for regulatory sandboxes, with a view to providing a level playing field.^126^ The Group also stated that a common testing framework would help cross-border trade, ‘thereby enhancing confidence in, and portability of, test outcomes to other European jurisdictions, and network effects by better and more formalised coordination between regulatory sandboxes’.^127^ Of course one must note that such benefits would be limited to addressing the sandbox as a stopgap solution, not the much bigger challenge of the regulatory framework within which FinTech operators must operate.

On paper a harmonised framework for regulatory sandboxes could help reduce FinTech forum shopping in relation to new FinTech models. That said, there are considerable logistical challenges to pursuing a harmonisation agenda for regulatory sandboxes, however well-intentioned. By definition sandbox regimes have to incorporate a certain amount of latitude—of necessity much has to come down to common sense exercise of discretion and judgement by regulators rather than rigid application of hard and fast rules. This makes defining a common approach a more delicate exercise than would usually be the case. A peculiarity of the regulatory sandbox is that regulatory sandboxes differ widely in legal form. Some are informally established with rules set out on a regulator’s website, while others involve a formal legislative act.^128^ This in itself creates challenges to the EU moving towards a formal legal set of rules.

More fundamentally, the bigger picture context is that regulatory sandboxes do not operate in a vacuum; they are an overlay on a non-negotiable financial services regulatory framework. A playing field for FinTech that would be comfortably level needs many more components than an EU-wide understanding of regulatory sandboxes. A harmonised legal framework for regulatory sandboxes in the EU would not address the regulatory patchwork effect created by the underlying regulatory gaps and the unfinished regulatory policy work that provide the *raison d’être* of the regulatory sandbox. Nor would it address cross-Member State regulatory friction on treatment of FinTech. However, even an imperfect expedient solution facilitating ground-breaking FinTech innovation to get off the ground may constitute a valuable stopgap. Any EU-specific perspective must also begin to encompass a post-Brexit perspective as the United Kingdom, the country that initiated the regulatory sandbox, forges its own independent path, and provides a competitor forum of choice for many innovators.

### The Challenge of Achieving an EU Cross-Border Regulatory Sandbox Regime

Within the context of discussion of the potential for a harmonised framework applicable to national regulatory sandboxes in operation, it is opportune to also weigh up the possibility of establishing cross-border, multilateral sandboxes within the EU. The availability of a co-ordinated one-stop shop approach to enabling cross-border testing could add to the attractiveness of the EU for FinTech companies.^129^ At present availing of a regulatory sandbox in existence in a Member State will only aid market entry in the relevant national territory (and indeed only in relation to the particular sectoral regulator unless a multi-agency approach becomes utilised). This may leave FinTech innovators who have participated in a sandbox more or less at square one in other Member States. As the ESAs perceptively note:

firms who have successfully tested innovations in a regulatory sandbox may face practical barriers to the application of these innovations in other Member States. For instance, firms may have to enter into extensive dialogue to explain again the concept of the innovation and measures to mitigate any risks, and may even be required to test again the proposition causing delays to roll-out.^130^

That being the case, an EU-wide or cross-border sandbox could be advantageous in enabling FinTech innovators to simultaneously approach market entry in more than one Member State thereby creating practical, resource and time efficiencies. For instance, this could be ideal for testing provision of cross-border payment services such as remittances. If multi-jurisdictional sandbox entry were permitted through the creation of a cross-border sandbox within the European Union, regulatory competition and the forum shopping calculations would then be diverted towards other competitive factors and regulatory differentiators in Member States such as the applicable tax regime. Although the details are scant, the Digital Finance Strategy places the onus on the EFIF to develop a procedural framework for cross-border testing in the EU with input from sandbox regulators.

The creation of a multilateral or cross-border sandbox would nonetheless be complex to achieve agreement on. Formulating how multifaceted applications to more than one national sandbox should be structured and processed would be thorny to resolve, as is evident from the Global Financial Innovation Network’s experience with an initial test-run of a multilateral sandbox.^131^ As a preliminary matter, a decision would need to be made as to whether applications to an EU cross-border sandbox would be processed on a centralised or decentralised basis. Delivering a cross-border EU sandbox would require a huge amount of goodwill and co-operation between national competent authorities. Such a development could potentially support the realisation and scaling up of FinTech innovation. However, Member States would have to be willing to co-operate, bearing in mind that in a fully decentralised model where a cross-border regulatory sandbox application could be made to any designated authority at Member State level, there would be no guarantee of keeping the slice of the FinTech pie within their Member State even if the initial approach and application was made there. Consequently establishing a centralised EU authority for receiving cross-border applications may be a more sensible and streamlined approach if the plunge is taken on cross-border sandboxes.

A first logical jumping off point could lie in the ESAs developing guidance to ensure a common approach for national competent authorities entering into bilateral or multilateral co-operation agreements in relation to regulatory sandboxes.^132^ This would stop short of realising a pan-EU sandbox. However, such co-operation when allied with progress towards a harmonised approach to regulatory sandbox standards may in turn lead to the establishment of a cross-border regulatory sandbox. Indeed there are some policy signals that favour this step being taken. In 2019 the potential for establishing an EU cross-border regulatory sandbox regime was floodlit by the specialist Expert Group on Regulatory Obstacles to Financial Innovation in its recommendation that the Commission and the ESAs should give further consideration to this option.^133^ Commission imprimatur beckoned in 2020 in the Digital Finance Strategy^134^ which contains a commitment to working with the European Forum of Innovation Facilitators^135^ to boost the work of national innovation hubs, but also crucially to providing a procedural framework that would allow cross-border testing and would allow firms to interact with supervisors from different Member States. It remains to be seen what that would look like.

Regardless of positive sentiments being expressed towards the prospects of an EU-wide regulatory sandbox, any investigation of a cross-border sandbox solution to enable new innovative products and services to test on a limited time and scale basis would need to robustly engage with the underlying EU regulatory topography. While it is the norm for domestic sandboxes to require an intention to provide the product or service within the territory of the sandbox, some revision of this would be needed for a truly pan-European sandbox operating across borders or providing services in more than one jurisdiction. A proportionality analysis would play a central role in a cross-border-European sandbox when interpreting and applying the financial market rulebook. An agreed understanding of proportionality and available regulatory discretion would be crucial to reach a common understanding concerning the appropriate level of inherent flexibility in the application of regulatory rules to sandbox participants. This would need to be appropriately calibrated.

Currently Member States with regulatory sandboxes are autonomously fashioning their own disparate regulatory approaches within the envelope of proportional accommodation provided by relevant EU financial services rules. For instance, the Dutch DNB (Central Bank) has stated that it regards the regulatory sandbox as offering latitude in the interpretation of the rules to ‘take our cue from the purpose of a rule, while we will also review established policies with new (technological) developments in mind and adapt these where necessary […] to accommodate innovation that actually contributes to our supervision objectives as much as practicable’.^136^ Meanwhile Hungary permits the creation of a tailor-made sandbox with relaxation of some domestic rules. Furthermore, its sandbox regime involves the creation of a preliminary licence for sandbox participants.

Undoubtedly the issues raised above present challenges. However, the biggest hurdle in the way of establishing a fully functioning cross-border EU sandbox regime may be principle-based objections from Member States. A known riposte from those who are anti-regulatory sandbox is that innovation hubs and FinTech helpdesks providing informal regulatory information are entirely sufficient. Although the regulatory sandbox has gained considerable traction, the majority of Member States do not have a regulatory sandbox. The concept of a regulatory sandbox is not universally liked by regulators in the Member States and remains controversial in influential Member States such as Germany and France. Fundamental objections may be voiced to the very concept of a regulatory sandbox and what it represents in terms of a regulator bending over to assist tech innovators, a stance removed from a more traditional conception of what it means to be a regulator or supervisor. Thus achieving sufficient Member State buy-in to any proposed EU-backed sandbox regime is far from certain. The FinTech Action Plan was cognisant of the fact that some competent authorities did not regard regulatory sandboxes with their competition-promotion aspect as within their mandate, while others zealously embraced the concept with a view to carving out FinTech turf.^137^ Some competent authorities have also questioned the two-tier approach created by regulatory sandboxes whereby sandbox participants receive tailored advice and monitoring while non-sandbox participants are effectively an out-group and subject to less preferential treatment.^138^

There may not be sufficient buy-in from Member States to feasibly achieve an EU cross-border sandbox regime allowing testing across multiple locations, particularly given that some regulators regard the sandbox as outside (or even opposed to) their mandate. A more sensible approach may therefore be to begin with a smaller scale means of dipping the EU’s toe in the water of experimental governance. Some EU trial initiatives in experimental governance that have begun to emerge in this space are discussed below.

### Alternative Paths: EU-Sanctioned Pilot Regulatory Regimes and Sectoral Sandboxes

As discussed above the majority of national regulatory sandboxes in the EU (and outside) are constructed to cover a broad range of FinTech applications. In contrast to this, emerging developments show that the Commission’s current comfort level sits at the incremental point of embrace of experimental governance on a sectoral basis. First, there is the ambition to launch a pan-European regulatory sandbox for blockchain by 2022. The Digital Innovation and Blockchain Unit in DG-CONNECT is co-operating with the European Blockchain Partnership (‘EBP’) on a regulatory sandbox that would permit testing applications of certain blockchain and digital assets.^139^ Secondly, within the Digital Finance Package, the Commission is championing a close cousin of the regulatory sandbox—the creation of a temporary EU legal melting pot for regulatory learning. Behind its Proposal for a Regulation on a pilot regime for market infrastructures based on DLT^140^ (the ‘DLT Pilot Proposal’) lies a recognition of the significance of applications of DLT in finance.^141^ The proposed Regulation is designed for certain investment firms, market operators and central securities depositories wanting to establish DLT market infrastructure and provide cross-border services throughout the EU. The objective is to provide an enabling framework to counteract the limited existing up-take of DLT in the context of backbone market infrastructures—trading venues and central securities depositories. As matters stand, regulatory obstacles and regulatory uncertainty abound amid a complex legal environment not designed with DLT-based business models in mind. Consequently, a key objective of enabling a pilot regime to allow experimentation to place is that it would assist in highlighting difficulties for secondary market trading of crypto-assets from the perspective of the existing EU framework for financial instruments. This would enable identification of what action could be taken by the EU to facilitate innovation by removing unjustifiable obstacles. At the same time, promotion of investor and consumer protection as well as market integrity and financial stability are expressed to be key objectives.^142^

In this study of evolving forms of adaptive regulatory strategies for FinTech, as a form of experimental governance, the EU’s DLT Pilot Proposal is striking. The DLT Pilot Proposal acknowledges the possibility of regulatory relaxation for participants, while to date national sandbox regulators have been largely hamstrung by the obligation to apply MiFID. By contrast, the DLT Pilot Proposal is pitched to ‘allow for experimentation through derogations for the use of DLT in the trading and post-trading of crypto-assets that qualify as financial instruments, where existing legislation precludes or limits their use’.^143^ This is radical in its express contemplation of the power to water down the application of regulatory requirements. Notably, in providing for sectoral derogations to be available for DLT multilateral trading facilities and DLT securities settlement systems from some aspects of financial services legislation, this would allow participants in the Pilot to benefit from a preferential regulatory regime as compared with that available to non-participants, something which sandbox regimes have generally assiduously tried to avoid.^144^

The planned DLT pilot regime is not being billed as a sandbox. Nor should it be. The DLT Pilot Proposal clearly traverses well beyond the usual understanding of sandboxes as firmly rooted in the applicability of financial services law to participants at all times both during and after sandbox participation. Rather, the DLT Pilot Proposal can properly be characterised as a more formal class of experimental governance whereby it is envisaged that hard law is used to create a temporary exemption regime through empowering legislative derogations. Experimental clauses of this kind have been advocated by the Council to encourage innovation.^145^ It is proposed that the special DLT trading regime be made available on an opt-in basis for up to six years and would be subject to an application process to the competent authority to ensure that there is no threat to financial stability, market integrity or investor protection. In common with sandbox regimes, the ‘test and learn’ element is present in the DLT Proposal. The objective is both to remove hurdles that exist for the issuing, trading and settlement of crypto-asset financial instruments and also to enable regulators to gain experience of handling how DLT works in such an environment.^146^ The expectation is that the DLT Pilot Proposal will allow ESMA and national competent authorities to become suitably familiar with DLT trading and associated risks. Although the fulfilment of this Proposal is inchoate, its arrival shows an EU penchant for experimental governance on a sector-specific scale. In relation to interacting with the wider landscape for regulatory sandboxes in the EU, it remains to be seen how far the EU is willing to go. In the meantime, a variety of alternative paths of experimental governance offer valuable learning opportunities for both institutional and regulatory actors in the FinTech space.

## Conclusion

This is an exciting time for digital finance policy development in all its forms in the Union as the MiCA proposal begins its journey. Regulatory fragmentation and level playing field problems act as disenablers to appropriate realisation of FinTech in the European Union. While regulatory inertia has been shaken off in favour of willing regulatory engagement and responsiveness to the FinTech agenda, much remains to play for. Even as relevant EU regulatory initiatives emerge to unify regulatory barriers to entry, the FinTech ecosystem continues to need support as it evolves to develop to its full potential. From a broader regulatory perspective, the regulatory sandbox is a FinTech enabler. For now, the regulatory sandbox provides a functional life-line for both FinTech innovators and regulators. As such, it demands EU regulatory attention. To some extent regulators who have jumped on the bandwagon have been fashioning loosely formed parameters as they go along. As highlighted by the analysis undertaken here of practices in Member States, there are marked divergences of approach and varying degrees of transparency across Member States that cry out for quality control and co-ordination on objective criteria to avoid undesirable market distortions around equality of access. Having conducted a wealth of information gathering and stakeholder dialogue, the EU now stands at a crossroads in terms of determining how to address the growing use of regulatory sandboxes by Member States to help FinTech innovators get over the line to full scale launch.

The regulatory sandbox has gained considerable traction across the European Union precisely because it neatly bridges the regulatory chasm of not having a business-friendly domestic or EU legal regime for FinTech. It allows regulators to assist in resolving the challenge presented by the unclear application of existing laws to impactful new technological applications. The fascinating wider picture is that the regulatory sandbox has the potential to yield benefits far beyond the outcomes for individual sandbox participants. Sandbox regulators in the EU can use the process, along with industry dialogue opportunities and innovation supports, to gain practical insights into how innovative technological applications are transforming the delivery of financial services. The working knowledge thus gleaned by regulators may prove invaluable in informing future policy choices concerning positioning the regulatory perimeter for FinTech activities.

The regulatory sandbox has earned its place. While acknowledging that not all regulators and Member States are on board with regulatory sandboxes, looking to the single market agenda, stakeholder dialogue should now lead at the least to the adoption of EU guidelines providing best practice indicators for operating national regulatory sandboxes in the European Union. Identifying best practice guidelines for incubation within Member State sandboxes would have a beneficial impact, not just for existing sandbox regulators within the EU, they would also transmit useful base-line messages on best practice to those jurisdictions where regulatory sandboxes remain at either design or review stage. Monitored operation of such guidelines on the operation of regulator sandboxes in Member States would help to achieve broad alignment on regulatory sandboxes where they are a chosen *modus operandi* while not compelling any Member State to provide a regulatory sandbox, thus preserving regulator autonomy. As highlighted, a legally harmonised framework for the operation of regulatory sandboxes would be challenging to achieve but could reduce cross-border dissonance. A less formal cross-border framework would still be very complex to develop but is worth the EFIF’s effort. Finally, the Commission’s unique DLT Pilot Proposal exemplifies a third way: a legislatively sanctioned, sector-specific, time-limited preferential regulatory regime to encourage market development.

Reflecting on existing and potential developments in the round, the ball lies in the court of the Commission as to how it will definitively address the dilemma of how much to respond to the regulatory sandbox phenomenon beyond a watching brief. Realistically, it may be that the Commission’s preference will not be to step up to formally act to regulate the regulatory sandbox offerings proliferating at Member State level. Rather, sector-specific pilot regimes in the model of the one proposed for DLT used in the trading and settlement for crypto-assets, combined with simultaneously encouraging the EFIF’s bottom-up development of a cross-border testing regime may represent the Commission’s preferred leadership path in experimental governance amid regulatory transition.

## Footnotes

1 The Financial Stability Board defines FinTech as ‘technologically enabled financial innovation that could result in new business models, applications, processes or products with an associated material effect on financial markets and institutions and the provision of financial services’. https://www.fsb.org/work-of-the-fsb/financial-innovation-and-structural-change/fintech/. Accessed 21 February 2021.

2 Given its many unique features as a clever, responsive approach to facilitating innovation, law and technology scholars have begun to devote attention to the regulatory sandbox as a fascinating regulatory adaption. See, e.g., Ahern (2019); Allen (2019); Buckley et al. (2020); Mangano (2018); Zetzsche et al. (2017).

3 European Commission (2020c); Council of Ministers (2020); Parenti (2020).

4 Hendrikse, Bassens and Van Meeteren (2018).

5 Payment Services Directive II (EU) 2015/2366 [2015] OJ L 337/35.

6 ‘Digital upstarts muscle in on US core banking service’, *Financial Times*, 26 September 2019, at https://www.ft.com/content/d74fbfd4-940b-11e9-8ff4-699df1c62544. Accessed 21 February 2021.

7 On relevant supervisory issues in relation to InsurTech see International Association of Insurance Supervisors (2017); International Association of Insurance Supervisors (2018). See also generally Lin and Chen (2020); Manes (2020).

8 Milne and Parboteeah (2016); Ahern (2018); Zetzsche and Preiner (2018).

9 There is no agreed definition of crypto-assets or their categories. The European Commission’s proposed definition of a crypto-asset in European Commission (2020d), Art. 3 of the Proposal for a Regulation on Markets in Crypto-Assets (‘MiCA’) is ‘a digital representation of value or rights which may be transferred and stored electronically, using distributed ledger technology or similar technology’. The Financial Stability Board defines a crypto-asset as ‘a type of private asset that depends primarily on cryptography and distributed ledger or similar technology as part of their perceived or inherent value’: Financial Stability Board (2019), p. 10. See generally Blandin et al. (2019).

10 See generally Baker and Dellart (2018); Chia (2020); Maume (2019); Lee (2020).

11 For a broad treatment of the implications of large scale and highly concentrated financial operating systems see Zetzsche et al. (2020).

12 See generally Pollman (2019).

13 The collapse in 2020 of Wirecard (Germany’s payment services giant) amid a large-scale accounting fraud represents a regulatory wake-up call in relation to the need for tightened governance requirements such as those applicable to banks.

14 On this see Brummer and Yadev (2019); Chiu (2019); Scherer (2016).

15 Ahern (2018).

16 Fleischer (2010), p. 236.

17 European Commission (2018); European Commission (2020a); European Commission (2020b); European Commission (2020c).

18 European Commission (2017).

19 European Commission (2018).

20 European Commission (2017), Priority Action 4.

21 Regulation (EU) 2020/1503 of the European Parliament and of the Council of 7 October 2020 on European crowdfunding service providers for business, and amending Regulation (EU) 2017/1129 and Directive (EU) 2019/1937 [2020] L 347/1.

22 When specified thresholds are exceeded for equity-based crowdfunding, the Prospectus Regulation (EU) 2017/1129 [2017] OJ L 168/12 will instead apply.

23 (EU) 2020/1504 [2020] OJ L 347/50.

24 MiFID II Directive 2014/65/EU on Markets in Financial Instruments [2014] OJ L 173/349 and MiFIR: the Markets in Financial Instruments Regulation 600/2014/EU [2014] OJ L 173/84.

25 European Commission (2020c).

26 Hofmann (2020).

27 The Central Securities Depositories Regulation (‘CSDR’) framework is ill-fitting for the arrival of tokenised securities. See Regulation (EU) No. 909/2014 of the European Parliament and of the Council of 23 July 2014 on improving securities settlement in the European Union and on central securities depositories and amending Directives 98/26/EC and 2014/65/EU and Regulation (EU) No. 236/2012 [2014] OJ L 257/1.

28 In a US context see Alkadri (2018); Brummer (2015a). In an EU context see Boreiko et al. (2019).

29 Hacker and Thomale (2018); Todorof (2019); Zilioli (2020).

30 Xie (2019).

31 Virtual Financial Assets Act 2018, Part II.

32 A full consideration of the proposed treatment of crypto-assets lies beyond the scope of this article.

33 European Commission (2020d). See also European Commission (2020f), the DLT Pilot Proposal discussed in Sect. 4 below.

34 As defined in Art. 4(1)(15) of MiFID II. See generally Sheridan (2017). MiCA is also designed to regulate the provision of crypto-asset services.

35 There are, however, unfortunate divergences of approach at a national level to the application of the MiFID II definition of ‘financial instruments’ to tokens such as Bitcoin. See generally Maas (2019).

36 As defined in Art. 2(2) of the E-Money Directive 2009/110/EC [2009] OJ L 267/7 (as amended by Directive (EU) 2015/2366) (‘EMD2’).

37 In addition, deposits within Art. 2(1)(3) of the Deposit Guarantees Directive 2014/49/EU [2014] L 173/149, structured deposits within Art. 4(1)(43) of MiFID II and Art. 2(1) of the Securitisation Regulation (EU) 2017/2402 [2017] OJ L 347/35 also fall outside the ambit of MiCA.

38 Although it is not contemplated that other types of crypto-asset be subject to an authorisation requirement, other regulatory requirements such as disclosures are provided for in MiCA in the interests of investor protection.

39 European Commission (2020c).

40 Regulation (EU) No. 910/2014 on Electronic Identification and Trust Services for Electronic Transactions in the Internal Market [2014] OJ L 257/73.

41 Regulation (EU) 679/2016 on the protection of natural persons with regard to the processing of personal data and on the free movement of such data (General Data Protection Regulation) [2016] OJ L 119/1. See also the Data Protection Law Enforcement Directive: Directive (EU) 2016/680 of the European Parliament and of the Council of 27 April 2016 on the protection of natural persons with regard to the processing of personal data by competent authorities for the purposes of the prevention, investigation, detection or prosecution of criminal offences or the execution of criminal penalties, and on the free movement of such data [2016] OJ L 199/89.

42 Directive (EU) 2018/843 of the European Parliament and of the Council of 30 May 2018 amending Directive (EU) 2015/849 on the prevention of the use of the financial system for the purposes of money laundering or terrorist financing, and amending Directives 2009/138/EC and 2013/36/EU [2018] OJ L 156/43 (‘AML5’).

43 Indeed, GDPR and AML requirements will receive further EU attention under the Digital Finance Strategy: European Commission (2020c).

44 The Commission held a public consultation on improving resilience against cyberattacks in financial services: European Commission (2019). This informed the development by the Commission of the Digital Operational Resilience Act (‘DORA’) legislative proposal: Proposal for a Regulation on digital operational resilience for the financial sector and amending Regulations (EC) No. 1060/2009, (EU) No. 648/2012, (EU) No. 600/2014 and (EU) No. 909/2014, COM(2020) 595 final.

45 Directive (EU) 2015/849 on preventing the use of the financial system for money laundering on terrorist financing [2015] OJ L 141/73 (‘AML4’); Regulation (EU) 2015/847 on information on the payer accompanying transfers of funds [2015] OJ L 141/1—makes fund transfers more transparent, thereby helping law enforcement authorities to track down terrorists and criminals; AML5.

46 Ahern (2019).

47 If the testing phase is promising, the applicant must then decide whether to go to a full launch (subject to obtaining any necessary regulatory authorisations).

48 Regulatory sandboxes are in operation in many developed countries outside the EU including Australia, Canada, Hong Kong, Japan, Norway, Singapore, Switzerland, the United Arab Emirates, and the United States.

49 For a good discussion of the global context see Buckley et al. (2020).

50 In January 2019 this was noted to include 21 EU Member States and 3 EEA States: European Supervisory Authorities (2019), p. 1.1.1.

51 Zetzsche et al. (2017).

52 Pursuant to an enabling amendment to the *Finanzmarktaufsichtsbehördengesetz* (Austrian Financial Authority Act), which entered into force on 1 September 2020.

53 FT Lab, https://www.dfsa.dk/Supervision/Fintech/FT-lab. Accessed 21 February 2021.

54 https://www.mnb.hu/en/innovation-hub/. Accessed 21 February 2021.

55 https://www.fktk.lv/en/licensing/innovation-and-fintech/innovation-sandbox/. Accessed 21 February 2021.

56 https://www.lb.lt/en/regulatory-sandbox. Accessed 21 February 2021.

57 https://www.mfsa.mt/fintech/regulatory-sandbox/. Accessed 21 February 2021.

58 https://www.dnb.nl/en/sector-information/supervision-stages/prior-to-supervision/innovationhub-and-regulatory-sandbox/regulatory-sandbox/. Accessed 21 February 2021.

59 The Spanish DTFS Law was passed on 5 November 2020 and the establishment of the sandbox is pending.

60 https://www.knf.gov.pl/en/MARKET/Fintech/Regulatory_Sandbox. Accessed 21 February 2021. Poland has an interesting public-private model planed for running its regulatory sandbox. The regulatory sandbox is under the remit of Poland’s financial supervisory authority (KNF) but a number of banks and other industry players provide the actual test bed environment.

61 On the nature of experimental governance see, for example, Sabel and Zeitlin (2012).

62 For example, authorisation requirements may be triggered by financial instruments including those from MiFID II.

63 Expert Group on Regulatory Obstacles to Financial Innovation (2019), p 70.

64 Accordingly, an argument can be advanced in relation to equity of access to the sandbox environment: Ahern (2019), p 360.

65 UNSGSA FinTech Working Group and CCAF (2019), p 32.

66 Google has obtained a banking licence in Ireland and an e-money licence in Lithuania.

67 In Germany this partnering arrangement is required to minimise risk and a regulatory sandbox is not provided. In France such partnering arrangements between FinTech start-ups and financial institutions are also common.

68 ‘Make proposed regulatory sandbox inclusive, fintech experts tell RBI’, *The Economic Times*, 4 June 2019, https://economictimes.indiatimes.com/small-biz/startups/newsbuzz/make-proposed-regulatory-sandbox-inclusive-fintech-experts-tell-rbi/articleshow/69644413.cms. Accessed 21 February 2021.

69 For example, Nivaura, an entity working on an Ethereum-based platform to issue equity tokens as a means of raising capital.

70 Corporations (FinTech Sandbox Australian Financial Services Licence Exemption) Regulations 2020, p 8(b) and/or the National Consumer Credit Protection (FinTech Sandbox Australian Credit Licence Exemption) Regulations 2020, p 8(b).

71 The FCA in the UK has a separate digital sandbox regime for products and solutions that are under development and are not ready for testing.

72 MNB (Hungary), Financial innovation testing environment licensing guide, p 2.4.

73 ASIC Corporations (Concept Validation Licensing Exemption) Instrument 2016/1175, and ASIC Credit (Concept Validation Licensing Exemption) Instrument. See also Monetary Authority of Singapore (2018, 2020).

74 Bank Negara Malaysia (2016).

75 Monetary Authority of Singapore (2020); Monetary Authority of Singapore, ‘MAS launches sandbox express for faster market testing of innovative financial services’, 7 August 2019, at https://www.mas.gov.sg/news/media-releases/2019/mas-launches-sandbox-express-for-faster-market-testing-of-innovative-financial-services. Accessed 21 February 2021.

76 It is notable that the UK’s FCA, a well-resourced and respected regulator, has not chosen the expedited decision-making route.

77 Malta offers either a six months or a 12 month testing period.

78 As seen in Australia, Indonesia, Mauritius and Thailand.

79 Outside the EU see for example, the State of Arizona, United States and Australia.

80 This is also the approach under the multilateral Canadian Securities Administrators’ regulatory sandbox, at https://www.securities-administrators.ca/industry_resources.aspx?id=1588. This may also be the case for the Hong Kong Monetary Authority’s FinTech Supervisory Sandbox, at https://www.hkma.gov.hk/eng/key-functions/international-financial-centre/fintech/fintech-supervisory-sandbox-fss/, and the Insurance Authority of Hong Kong’s Insurtech Sandbox, at https://www.ia.org.hk/en/aboutus/insurtech_corner.html. No information on standard duration is provided on the respective webpages. All accessed 21 February 2021.

81 For example, the Bank of Lithuania and the Financial and Capital Market Commission (FCMC) in Latvia offer the possibility of an extension of up to 12 months.

82 https://www.dfsa.dk/~/media/Fintech/Selection_criteria.pdf?la=en. Accessed 21 February 2021.

83 There is nonetheless an unfortunate risk that competitive acceptance to a regulatory sandbox will be prematurely construed by the public as a seal of approval of the firm and its FinTech offering by the relevant sandbox regulator. On this see Ahern (2019), p 362; Financial Stability Institute (2020), p 122.

84 The Capital Requirements Directive IV 2013/36/EU [2013] OJ L 176/338 and Capital Requirements Regulation (EU) 575/2013 [2013] OJ L 176/1 (‘CRD IV’).

85 For example, the EIOPA Guidelines on outsourcing to cloud service providers: European Insurance and Occupational Pensions Authority (2020).

86 Bank Negara Malaysia (2016), p 8.5.

87 Siebert and Koop (1993).

88 On this notion in economics see Apolte (2002).

89 Sun and Pelkmans (1995).

90 Siebert and Koop (1993), p 20.

91 Ahern (2018).

92 European Commission (2020f).

93 European Commission (2020c).

94 European Commission (2018).

95 European Commission (2020a), p 3.

96 European Supervisory Authorities (2019).

97 European Commission (2018).

98 European Supervisory Authorities (2019).

99 https://esas-joint-committee.europa.eu/Pages/Activities/EFIF/European-Forum-for-Innovation-Facilitators.aspx. Accessed 21 February 2021. ‘Innovation facilitators’ is used as an umbrella term to cover both innovation hubs and regulatory sandboxes.

100 ‘Financial technology: European Commission and European Supervisory Authorities launch new platform to improve cooperation on technological innovation in the financial sector’, 2 April 2019, at https://ec.europa.eu/commission/presscorner/home/en. Accessed 21 February 2021.

101 See Sect. 4.4 below on the expected future contribution of EFIF to a framework for cross-border testing.

102 De Burca and Scott (2006); Eberlein and Kerwer (2004); Trubek and Trubek (2007).

103 European Supervisory Authorities (2019), p 119.

104 European Commission (2018), p 7.

105 On the increasing use of soft law and new governance mechanisms in EU law see Terpan (2015).

106 European Commission (2018), at 1.3.

107 Sabel and Zeiltin (2008).

108 Brummer (2015b); Schemmel (2016). For a general discussion of the emergence of ESMA as the pre-eminent supervisory agency see Gortsos and Lagaria (2020).

109 Securities and Markets Stakeholders Group (2018), p 54.

110 European Supervisory Authorities (2019).

111 Council of Ministers (2020).

112 Gortsos and Lagaria (2020).

113 European Commission (2014), section 3.

114 European Commission (2020c).

115 Regulation (EU) 2019/2175 of the European Parliament and of the Council of 18 December 2019 amending Regulation (EU) No. 1093/2010 establishing a European Supervisory Authority (European Banking Authority), Regulation (EU) No. 1094/2010 establishing a European Supervisory Authority (European Insurance and Occupational Pensions Authority), Regulation (EU) No. 1095/2010 establishing a European Supervisory Authority (European Securities and Markets Authority), Regulation (EU) No. 600/2014 on markets in financial instruments, Regulation (EU) 2016/1011 on indices used as benchmarks in financial instruments and financial contracts or to measure the performance of investment funds, and Regulation (EU) 2015/847 on information accompanying transfers of funds [2019] OJ L 334/1.

116 Expert Group on Regulatory Obstacles to Financial Innovation (2019), p 70.

117 On the question of sandbox regulators as competition promoters in FinTech markets see Ahern (2019); Zetzsche et al. (2017).

118 Securities and Futures Commission Regulatory Sandbox, at https://www.sfc.hk/web/EN/sfc-fintech-contact-point/sfc-regulatory-sandbox.html. Accessed 21 February 2021.

119 Insurance Authority (Hong Kong), Insurtech Sandbox, at https://www.ia.org.hk/en/aboutus/insurtech_corner.html#1.

120 https://www.securities-administrators.ca/industry_resources.aspx?id=1626. Accessed 21 February 2021.

121 Canadian Securities Administrators, CSA Regulatory Sandbox, at https://www.securities-administrators.ca/industry_resources.aspx?id=1588. Accessed 21 February 2021.

122 European Supervisory Authorities (2019), p 97.

123 European Supervisory Authorities (2019), p 99–100.

124 European Commission (2020c).

125 European Supervisory Authorities (2019), Appendix B, p 19.

126 Expert Group on Regulatory Obstacles to Financial Innovation (2019), p 70.

127 Ibid., p 70.

128 For example, regulatory sandboxes in Spain and Italy have followed the formal route of an enabling legislative decree being passed.

129 Parenti (2020).

130 European Supervisory Authorities (2019), p 111.

131 Global Financial Innovation Network (2020).

132 European Supervisory Authorities (2019), paras. 114–115.

133 Expert Group on Regulatory Obstacles to Financial Innovation (2019), p 70.

134 European Commission (2020c).

135 The terms of reference of the European Forum for Innovation Facilitators expressly contemplate the possibility of agreeing joint regulatory sandbox testing arrangements.

136 AFM-DNB (2016).

137 European Commission (2018), at 1.3.

138 European Supervisory Authorities (2019), para. 101. See also Chiu (2017), p 759; McCarthy (2019).

139 European Commission (2020e). For a recommendation of a dedicated regulatory sandbox for robo-advice see Ringe and Ruof (2018).

140 European Commission (2020f).

141 On the potential of DLT for finance see generally Avgouleas and Kiayias (2019).

142 European Commission (2020f), p 2.

143 European Commission (2020f), p 3.

144 Ahern (2019).

145 Council of Ministers (2020).

146 European Commission Press Release, Brussels 24 September 2020, ‘Digital finance package: Commission sets out new, ambitious approach to encourage responsible innovation to benefit consumers and businesses’, https://ec.europa.eu/commission/presscorner/detail/en/IP_20_1684. Accessed 21 February 2021.
